# Emergence of *Coxiella burnetii* in Ruminants on Reunion Island? Prevalence and Risk Factors

**DOI:** 10.1371/journal.pntd.0003055

**Published:** 2014-08-07

**Authors:** Eric Cardinale, Olivier Esnault, Marina Beral, Florence Naze, Alain Michault

**Affiliations:** 1 Centre de coopération Internationale en Recherche Agronomique pour le Développement (CIRAD), UMR 15 CMAEE, Sainte Clotilde, La Réunion, France; 2 Institut National de la Recherche Agronomique (INRA), UMR 1309 CMAEE, Sainte Clotilde, La Réunion, France; 3 Centre de Recherche et de Veille sur les maladies émergentes dans l'Océan Indien (CRVOI), plateforme de recherche CYROI, Sainte Clotilde, La Réunion, France; 4 Groupement de Défense sanitaire de la Réunion, le Tampon, Réunion, France; 5 CHU de la Réunion, St Pierre, Réunion, France; University of California San Diego School of Medicine, United States of America

## Abstract

Q fever is a widespread zoonosis that is caused by *Coxiella burnetii* (*C. burnetii*), and ruminants are identified as the main sources of human infections. Some human cases have been described, but very limited information was available about Q fever in ruminants on Reunion Island, a tropical island in the Indian Ocean. A cross-sectional study was undertaken from March 2011 to August 2012 to assess the Q fever prevalence and to identify the major risk factors of *C. burnetii* infection in ruminants. A total of 516 ruminants (245 cattle, 137 sheep and 134 goats) belonging to 71 farms and localized in different ecosystems of the island were randomly selected. Samples of blood, vaginal mucus and milk were concomitantly collected from females, and a questionnaire was submitted to the farmers. Ticks from positively detected farms were also collected. The overall seropositivity was 11.8% in cattle, 1.4% in sheep and 13.4% in goats. *C. burnetii* DNA was detected by PCR in 0.81%, 4.4% and 20.1% in cow, sheep and goat vaginal swabs, respectively. *C. burnetii* shedding in milk was observed in 1% of cows, 0% in sheep and 4.7% in goats. None of the ticks were detected to be positive for *C. burnetii*. *C. burnetii* infection increased when the farm was exposed to prevailing winds and when there were no specific precautions for a visitor before entering the farm, and they decreased when a proper quarantine was set up for any introduction of a new ruminant and when the animals returned to the farm at night. MLVA genotyping confirmed the role of these risk factors in infection.

## Author Summary

Q fever is a disease that could be transmitted from animals (cattle, sheep and goats) to humans and caused by a bacterium called *Coxiella burnetii* (*C. burnetii*). Some human cases exhibiting characteristic clinical signs of that disease have been detected on Reunion Island, a tropical island in the Indian Ocean, but to date, we did not know if these animals could be seen as potential sources of the disease. Thus, a study was undertaken from March 2011 to August 2012 to detect the presence of that bacterium in these animals and to understand how they could get infected themselves. A total of 516 ruminants (245 cattle, 137 sheep and 134 goats) belonging to 71 farms and localized in different environments of the island were selected. Samples of blood, vaginal mucus and milk were concomitantly collected from females, and a questionnaire was submitted to the farmers. Ticks from positively detected farms were also collected. We observed 11.8% of cattle, 1.4% of sheep and 13.4% of goats had already been in contact with the bacterium. *Coxiella burnetii* was also directly detected in some vaginal and milk samples. None of the ticks were detected to be positive for *C. burnetii*. We found that the ruminants could be infected when their farm was exposed to prevailing winds because the bacterium can be transported by the wind, and when there were no specific precautions for visitors before entering the farm, because they could act as mechanical carriers of *Coxiella*. Conversely, keeping new animals under surveillance for some days to detect any signs of the disease before they enter the farm or keeping the animals in the barn at night limit the risk of infection.

## Introduction

Q fever is a widespread zoonosis that is caused by *Coxiella burnetii* (*C. burnetii*), an obligate intracellular bacterium [1]–[4]. The reservoir includes mammals, birds and arthropods, mainly ticks [5]. Ruminants (sheep, goats and cattle) are identified as the main sources of human infections [6], [7]. Humans are infected mainly by inhalation of an aerosol contaminated with parturient products from the urines or feces of infected animals [8].

The risk of transmission of *C. burnetii* is dependent on the prevalence of shedder ruminants and on the level of shedding. *C. burnetii* is shed by ruminants mainly by birth products, but it may be shed via the vaginal mucus, milk, feces, urine and semen [9]. To control the spread of *C. burnetii* among animals as well as from animals to humans, the detection of shedders of *C. burnetii* and the knowledge of the prevalence of the infection are imperative. The risk of zoonosis also depends on the level of *C. burnetii* in the products of the infected animals.

Serological tests (complement fixation, indirect immunofluorescence and enzyme-linked immunosorbent assays (ELISA)) are classically used in epidemiological studies to detect carriers of antibodies against *C. burnetii*. Serological tests indicate previous exposure [10] to *C. burnetii* and are not appropriate for the identification of shedder ruminants, especially because seronegatives are present among them [11], [12]. This lack of sensibility in this technique is lower using ELISA [13].

Isolation of *C. burnetii* is not performed for epidemiological investigation because it is difficult, time consuming and requires confined level L3 laboratories. Conventional polymerase chain reaction presents a very useful method for the detection of *C. burnetii* DNA [9], [14]. The real-time PCR assays are now recognized as the most convenient tools because these tests have excellent sensitivity, specificity and permits investigators to obtain quantifiable information. Real-time PCR is adapted to large scale studies because this technique can be semi-automated, thus reducing the risk of sample contamination and permitting gained time.

Reunion Island is a French overseas department that has a population of approximately 800,000 inhabitants. Reunion Island is a hotspot in the Earth's crust located in the Indian Ocean, east of Madagascar, approximately 200 km south-west of Mauritius, the nearest island. The island is 63 km long and 45 km wide and covers an area of 2,512 km^2^. Cities are concentrated on the surrounding coastal lowlands. The climate is tropical and humid, with two main seasons: a hot rainy season from December to March, and a dry and cold season from April to November. The eastern coast (the “windward” coast) experiences rainfall of approximately 2,000 mm per year, whereas the western coast (the “leeward” coast) has an annual rainfall of less than 2,000 mm. The domesticated animal populations on the island comprise approximately 40,000 cattle, 30,000 goats and 2,000 sheep. To date, no information was available about Q fever in humans and animals.

The present study aimed to provide epidemiological information about Q fever in the animal population of Reunion Island using available diagnostic tools and appropriate samples. The data will be used to appreciate the prevalence of *C. burnetii* infection in the three main domestic ruminant species: cattle, sheep and goats at both the animal and herd levels, as well as to identify the major risk factors of infection.

## Materials and Methods

### Ethics statement

The research protocol was implemented with the approval of the Direction of Agriculture, Food and Forestry (DAAF) from the French Ministry of Agriculture, under the European animal welfare regulation (project license number 102498). No endangered or protected species were involved in the survey. All the farmers gave their permission to be included in the study and for the samples. The animals were sampled without suffering.

### Study design

#### Herds and animals investigated

From March 2011 to August 2012, a total of 516 ruminants (245 cattle, 137 sheep and 134 goats) belonging to 71 farms and localized in different ecosystems of Reunion Island were randomly selected for this study. The sample size was considered at a 95% level of confidence, 5% of desired absolute precision and expected prevalence of 10% for cattle, sheep and goats [15]. Only female animals were sampled. At least five cows were chosen from each cattle herd (46) and 10 goats and ewes were chosen from each small ruminant herd (25).

#### Data collection

The study was based on data taken from interviews with the farmers. Data concerning farm characteristics, type of production, number of animals, proximity to another farm or sugar cane fields, presence of organic and other waste within the farm, wind exposure, proximity to a permanent water source, stability type, presence of ticks on animals, use of treatment against ectoparasites and insects, contact of animals with other animals or humans, grazing practice, manure spread on pastures, presence of tenrecs, rodent control, occurrence of pregnancy terminations within the herd during the last 12 months, purchasing behavior, quarantine of newly purchased animals and other biosecurity related factors such as hygienic precautions taken by the staff or any other people entering the farm (truck driver, veterinary and other visitors) were collected using a questionnaire. This questionnaire was pre-tested in a preliminary study in five farms. The final questionnaire had 40 questions, 75% of which were close-ended.

### Laboratory analysis

#### Collection of samples

Samples of blood, vaginal mucus and milk were concomitantly collected from each selected ruminant. Vaginal mucus samples were taken from inside the vagina with a dry sterile swab and then place into a transport medium (Virocult). At least 2 ml of milk from the teats were sampled aseptically into a sterile container. Blood samples were obtained from a jugular vein and were collected into sterile Vacutainers.

One hundred and twenty nine ticks (all belonging to the Rhipicephalus genera) were collected from seven farms that exhibited positive PCR and put into sterile containers.

All of the samples were transported to the laboratory within 24 h of collection in a biosafety container at 4°C. Milk, vaginal samples and ticks were then frozen at −80°C for subsequent PCR. Blood was centrifuged (3000 g, 10 min), and sera were frozen at −20°C.

#### Serological technique

Sera were screened for the presence of *C. burnetii* IgG phases 1 and 2 specific antibodies by a commercial enzyme-linked immunosorbent assay (ELISA) according to the manufacturer‘s instructions (LSIVET Ruminant Milk/Serum Q Fever ELISA COXLS LSI, Lissieu, France). The antigen is a sheep strain (phase 1–2). Sensitivity of this ELISA test reaches 87% and specificity 100% (manufacturer's data).

#### Real time quantitative PCR assay

First, each tick was crushed in 500 µl of Lysis Buffer Nuclisens (BioMérieux, Marcy l'Etoile, France) by a still ball on a grinder (Retsch Fisher Bioblock Scientific, Illkirch, France) for 7 min (frequency of grinding 30/s).

Total nucleic acids were extracted from 500 µL samples or 400 µL of the homogenate supernatant from the ticks. Extraction was performed using the BOOM technology automated in the NucliSens easyMAG apparatus (BioMérieux, Marcy l'Etoile, France). Fifty-five µL of the magnetic silica was used per extraction.

PCR amplifications were performed using a LightCycler 480 system (Roche Diagnostics, Meylan, France) and LC 480 Probe Master kit (Roche diagnostics, Meylan, France).

The real-time Taqman PCR procedure used here targeted a fragment of the transposase gene located in the IS1111 genome region of *C. burnetii* [16]. The forward primer, Cox-F (5′-GTC TTA AGG TGG GCT GCG TG) and the reverse primer, Cox-R (CCC CGA ATC TCA TTG ATC AGC) amplifies a 295 bp fragment that was revealed by a TaqMan probe (FAM-AGC GAA CCA TTG GTA TCG GAC GTT TAT GG-TAMRA). These primers and probe were synthetized by Tib Mol-Biol (Tib Mol-Biol, Berlin, Germany).

Assay conditions were optimized using varying primers and probe concentrations, as well as different concentrations of DNA extracts. To evaluate the qualitative performance of the *C. burnetii* PCR assay, DNA was and diluted to 1/10, 1/100 and 1/1000, and 29 negative samples (milk or vaginal mucus) and 21 positive samples (milk or vaginal mucus) that were obtained from INRA Tours France were tested.

Routine PCR was performed in a 20 µL reaction volume. The 20 µL PCR mix for the detection of *C. burnetii* contained 4 µl of purified DNA, 0.15 nM of Cox-F primer, 0.3 nM of Cox-R and 0.10 nM of probe. The thermal cycling consisted of 95°C for 8 min, followed by 45 cycles at 95°C for 15 s and 60°C for 30 s. Each test run included a negative control and a positive control. The positive control (see below), the synthetic DNA, was used at a concentration near the lower limit of detection to optimize the detection system, yet high enough to provide consistent positive results. Positive and negative controls were co-extracted with samples.

The reference gene GAPDH was used as an internal control for nucleic acid extraction and amplification. The forward primer GAPDH-F (5′GAA GGT GAA GGT CGG AGT -3′) and the reverse primer GAPDH-R (5′-GAA GAT GGT GAT GGG ATT TC-3′) amplified a 226 bp fragment that was revealed by the TaqMan probe Fam-CAA GTC TCC CGT TCT CAG CC-Tamra. Primers and probes were synthetized by Tib Mol-Biol (Tib Mol-Biol, Berlin, Germany). The 20 µL PCR mix for the detection of the GAPDH gene contained 4 µl of purified DNA, 0.5 nM of GAPDH-F, 0.5 nM of GAPDH-R and 0.2 nM of probe. Thermal cycling conditions were similar to that of *C. burnetii* PCR.

#### Quantification using plasmid standard curves

A synthetic DNA fragment was used as an external standard for the absolute quantitation of the assay. The *C. burnetii* target sequence was amplified by PCR, purified using the GeneClean Turbo Kit (Qbiogene, Illkrich, France) and cloned into the T7 expression pGEM-T Easy (Promega, Lyon, France). The amount of DNA was estimated using the Quanti PicoGreen DNA assay Kit (Invitrogen, Cergy Pontoise, France) in the LightCycler 2.0 system.

Ten samples of each dilution of the synthetic DNA fragment were tested to determine the detection threshold. The detection threshold was the lowest RNA titer with 100% detection rate.

DNA diluted at 1/100 (INRA) was amplified ten times in the same run to evaluate intra-experimental reproducibility, and in ten different runs to evaluate inter-assay reproducibility.

#### VNTR genotyping

Two VNTRs (locus name: Cbu1435_ms33_7 bp_9U_262bp and Cbu1471_ms34_6 bp_5U_210bp) were used in the present study and were among the VNTRs tested and previously described by Arricau-Bouvery et al. [17]. The flanking primers used to amplify the markers are Cbu1435: forward TAG GCAGAG GACAGAGGACAGT, reverse ATGGATTTAGCCAGCGATAAAA and Cbu1471: forward TGACTATCAGCGACTCGAAGAA, reverse TCGTGCGTTAGTGTGCTTATCT.

PCR amplification was performed using 47 DNA from *C. burnetii* obtained from the vaginal mucus of goats. The final reaction volume of 20 µl contained 5 µl of extract, 0.3 µM concentration of each primer (Tib Mol-Biol, Berlin, Germany) 1 µl of Amplitaq Gold (Qbiogen Illrich, France), 1x PCR buffer (Invitrogen, Cergy Pontoise, France) and 200 µM of each deoxynucleotide triphosphate (Invitrogen, Cergy Pontoise, France). Amplifications were performed in a Gene Amp 9600 thermal cycler (Perkin Elmer, Courtabeuf, France). Initial denaturation at 94°C for 5 min was followed by 60 cycles of 94°C for 30 sec, 60°C for 30 sec, 70°C for 1 min. The final extension step was 5 min at 72°C.

Five microliters of amplification product was loaded on a 2% standard agarose gel (Eurobio, Courtabeuf, France). Gels were stained with SYBR Safe DNA gel stain (Invitrogen, Cergy Pontoise, France). The size marker used was a 100 bp low ladder (Sigma-Aldrich, Saint Quentin Fallavier, France). Gel was photographed under UV light and images were managed using the Photocapt Software package (Vilber-Lourmat, Torcy, France).

### Statistical analyses

The animals were considered positive when at least one sample (blood, swab or milk sample) tested positive by either serology or PCR. The serological and PCR data were analyzed using a generalized linear mixed model (glmmML library, R software), where the individual health status was the binomial response, and the variables from the questionnaire were the explicative factors.

All of the explicative variables were categorical. The number of categories per variable was limited, such that frequencies of categories were only >10%. These variables were selected from a preliminary step aimed at lowering the chance of obtaining results affected by multicollinearity in the dataset [18]. All bilateral relationships between these variables were evaluated (*χ*2). A two-stage procedure was used to assess the relationship between explanatory variables and the health status of the animals. Logistic regression was used according to the method described by Hosmer and Lemeshow [19]. In the first stage, a univariate analysis was performed to relate Q fever positivity to each explanatory variable. Only factors associated (Pearson *χ*2-test, *P*<0.25) with Q fever positivity were offered to a full model for multivariable analysis [20]. The second stage involved a logistic multiple-regression model. The contribution of each factor to the model was tested with a likelihood-ratio *χ*2 through a stepwise procedure (backward and forward). At the same time, the simpler models were compared to the full model by the Akaike information criterion [21]. This process was continued automatically until a model was obtained with all factors significant at *P*<0.*05* (two-sided). Goodness-of-fit of the final model was assessed using Pearson *χ*2, Deviance and the Hosmer–Lemeshow tests [19].

## Results

### Specificity and sensitivity

The evaluation of the specificity of the real time PCR assays was reported by Klee et al. (2006) [22]. In the present study, all negative samples from INRA were negative, confirming the specificity of the assays.

All of the samples that were found positive by INRA were confirmed positive. The detection threshold determined from dilution series of synthetic DNA showed that this PCR allowed the detection of 24 samples of 500 copies of the genome/mL and one sample of 250 *C. burnetii* particles/ml sample, the number of IS1111 elements in the genome being determined to be close to 20 for the Nine Mile strains [22].

### Intra and inter-assay reproducibility

Coefficients and Ct averages of intra- and inter-assays were 0.46% and 1.4%, 26.54 and 26.63, respectively.

### Quantification from infected animals

Bacterial load in vaginal samples by ml of transport medium ranged from 50,600 to 255,000 for cattle, from 82,400 to 314,000 for sheep and from 112,000 to 385,000 for goats.

### MLVA genotyping

The typability of the two loci was 85.1% (40 of 47 positive PCR for goats). We obtained nine genotypes among the 40 amplified DNA samples (Table 1).

**Table 1:** MLVA genotypes from goat vaginal swabs (8 farms, Reunion Island, 2011–2012).

						MLVA typing results	
Farm ID	Animal species	Approximate herd size	No. samples tested	Sample	No. Samples included in study	MLVA ID	No. samples
A	Goat	150	25	vaginal swabs	25	1	10
						2	2
						3	1
						4	4
						7	7
B	Goat	40	3	vaginal swabs	1	5	3
C	Goat	50	2	vaginal swabs	2	5	2
D	Goat	100	3	vaginal swabs	1	5	5
E	Goat	50	6	vaginal swabs	6	5	2
						7	2
						8	2
F	Goat	40	3	vaginal swabs	3	6	1
						7	1
						8	1
G	Goat	25	1	vaginal swabs	1	9	1
H	Goat	30	3	vaginal swabs	3	5	3

### Prevalence

**Figure 1:**
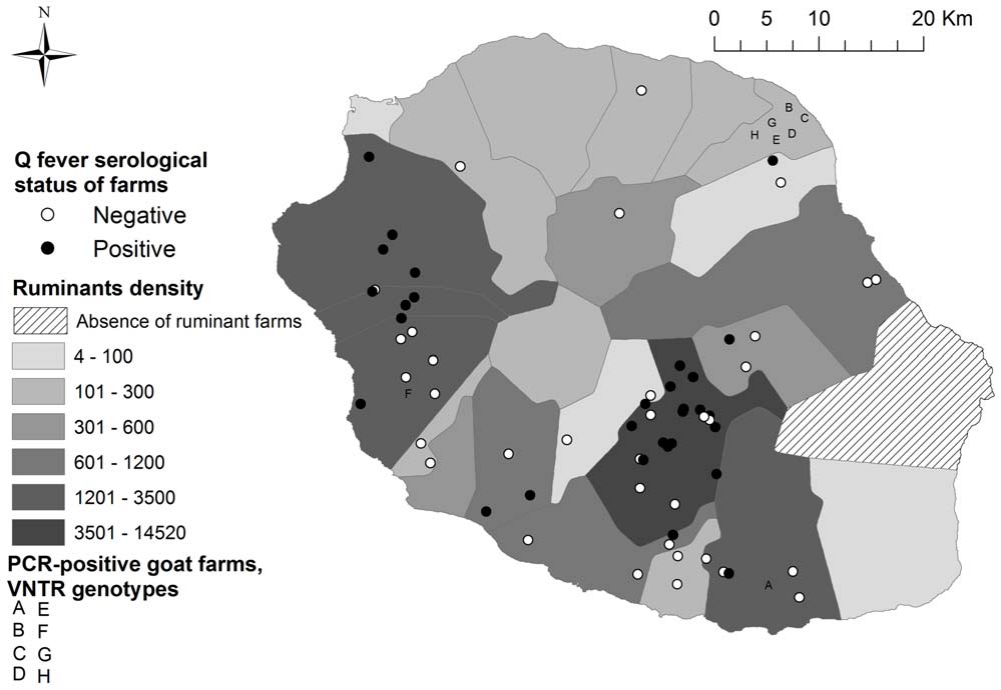
Q fever serological status of ruminant farms and detected MLVA genotypes (71 farms, 2011–2012; Reunion Island).

The overall seropositivity was 11.8% (95% CI 7.8 – 15.9) in cattle, 1.4% (95% CI 0 – 3.5) in sheep and 13.4% (95% CI 8.2–25.6) in goats. *C. burnetii* DNA was detected by PCR in 0.81% (95% CI 0–1.9) of cow vaginal swabs, 4.4% (95% CI 0.9 – 7.8) of ewe vaginal swabs and 20.1% (95% CI 13.3 – 26.9) of goat vaginal swabs. *C. burnetii* shedding in milk was observed in 1% (95% CI 0.2 –1.8) of cows, 0% in sheep and 4.7% (95% CI 0 – 11.2) in goats. Twenty-one out of 46 (95% CI 32 – 60) cattle farms were found to be positive either in serology or PCR, 50% (95% CI 33 – 67) of sheep farms and 41% (95% CI 18 – 64) of goat farms. All of these farms were spread throughout the island (figure 1). The within-herd prevalence in the positive farms ranged from 20% to 40% in cattle farms and from 30% to 90% in small ruminant farms. None of the ticks collected were detected to be positive for *C. burnetii*.

### Risk factors

After variable selection (Table 2), the logistic multiple-regression model indicated that the risk of *C. burnetii* infection was increased when the farm was exposed to prevailing winds (OR = 2,11; 95% CI [1,13; 3,99]) and when there were no specific precautions for a visitor before entering the farm (OR = 3,13; 95%CI [1,57; 6,70]), and decreased when a proper quarantine was set up for any introduction of new ruminant (OR = 0,06; 95%CI [0,01; 0,17]) and when the animals went back to the farm at night (0,53; 95%CI [0,42; 0,64]) (Table 3).

**Table 2:** Explanatory variables included in the analysis of *C. burnetii* infection (71 farms, Reunion Island, 2011–2012).

Variables		% of farms	% of positive farms	Animal seroprevalence	P-value
Renewal of ruminants population	No	16,22	33,33	0,293	-
	Yes	72,97	48,15	0,253	0.103
Proximity to another farm	No	17,57	46,15	0,245	-
	Yes	71,62	32,88	0,273	0.089
Exposure to dominant winds	No	44,59	42,42	0,238	-
	Yes	55,41	39,02	0,253	0.08
Animal grazing	No	29,73	18,18	0,215	-
	Yes	70,27	48,15	0,253	0.068
Treatment against ectoparasites	No	31,08	21,74	0,182	-
	Yes	68,92	49,02	0,253	0.016
Animals kept in the farm at night	No	48,65	44,44	0,273	-
	Yes	51,35	36,84	0,241	0.109
Environmental control	No	36,49	22,22	0,296	-
	Yes	63,51	51,06	0,246	0.022
Martin (*Acridotheres tristis*) presence on the ruminants	No	70,27	34,62	0,22	-
	Yes	29,73	54,55	0,296	0.091
Access to a water point on the pasture	No	66,22	40,82	0,265	-
	Yes	32,43	40,00	0,253	0,121
Antibiotic treatment after abortion	No	72,97	31,48	0,241	-
	Yes	27,03	65,00	0,241	0.097
No specific protection for a visitor entering the farm	No	54,05	22,50	0,097	-
	Yes	45,95	61,76	0,138	2.81.10-7
Quarantine	No	56,76	64,29	0,135	-
	Yes	43,24	9,38	0,123	4.210.10-7
Sale and purchase of animals	No	47,30	11,43	0,104	-
	Yes	52,70	66,67	0,134	6.100.10-5

**Table 3:** Final logistic regression model for risk factors for *C. burnetii* infection of ruminants, Reunion Island (71 farms; 2011–2012).

Variables		Estimate*	SE (standard error)	*P*-value	Odds Ratio and 95% CI
Exposure to winds	No	-	-	-	-
	Yes	0.747	0.321	0.019	2.11 [1.13; 3.99]
Animals kept in the farm at night	No	-	-	-	-
	Yes	−0.641	0.319	0.044	0.53 [0.01; 0.77]
Quarantine	No	-	-	-	-
	Yes	−2.838	0.616	4.18.10^−6^	0.06 [0.01; 0.17]
No specific protection for a visitor entering the farm	No	-	-	-	-
	Yes	1.141	0.366	1.84.10^−3^	3.13 [1.57; 6.70]

*Intercept =  -3.18; Model deviance = 44.25; AIC = 116.71, model df = 7 (p<0,001).

## Discussion

To the best of our knowledge, this is the only documented epidemiological study on Q fever in ruminants in Reunion Island, highlighting that *Coxiella burnetii* is endemic in this territory.

For our epidemiological survey, we used both serological and PCR techniques to better understand the characteristics of Q fever. Complement fixation technique remains widely used by laboratories in many countries to assess the seroprevalence of *C. burnetii* infection. This method yields good results for routine diagnosis at the herd level, but multiple studies have concluded (World Organisation for Animal Health 2010) that CFT is less sensitive than ELISA testing. Following international suggestions, ELISA results are deemed reliable for the screening of seroprevalence [23], [24]. However, serological tests (complement fixation or ELISA) only detect antibody-carriers against *C. burnetii*, demonstrating the previous exposure to the pathogen but not the current shedding of the pathogen [10]. Because we aimed to assess the overall pattern and characteristics of Q fever in Reunion island, the detection of shedders of *C. burnetii* was important because they are one of the critical points for the control of spreading of the bacteria among animals and from animals to humans [3]. Polymerase chain reaction (PCR) has been used to detect *C. burnetii* DNA in biological samples. Additionally, we employed a real-time PCR technique that is currently being developed with the aim of providing quantifiable information. The technique allows a priori scaling in the importance of sources of bacterium with regards to the risk of transmission of *C. burnetii* among animals and from animals to humans. Finally, on the contrary to conventional PCR, real-time PCR can be automated, leading to both a lower risk of sample contamination and a more time-efficient method of detection [25]. Because we found a bacterial load 40 to 310 times higher than the detection threshold, the probability for false negatives remained low. Finally, we observed nine different MLVA genotypes with very good typability compared to that obtained from Roest et al. (53%) [26], possibly due to the number of cycles (60).

In our study, the overall seropositivity was 11.8% in cattle, 1.4% in sheep and 13.4% in goats. These results are much lower than those observed in Europe; for example, ELISA testing showed 38.0% in cattle and 6.0% in sheep for individual seropositivity in Hungary [27]. In Northern Spain, ELISA anti-*C. burnetii* antibody prevalence was slightly higher in sheep (11.8±2.0%) than in goats (8.7±5.9%) and beef cattle (6.7±2.0%) [28]. Our seroprevalence rates were also lower compared to the results from other tropical countries. The seroprevalence in cattle was estimated to be between 40% and 59.8% in Nigeria, Sudan and Zimbabwe, and only 4% Chad [29]. The seroprevalence in sheep has been reported to vary between countries: 62.5% in Sudan, 22.5% in Egypt and 11% in Chad. Additionally, differences were also observed for seroprevalence in goats: 53% in Sudan, 16.3% in Egypt and 10% in Zimbabwe [29]. Our PCR results were quite surprising, with a low prevalence of *C. burnetii* in cow and ewe vaginal swabs (0.81% and 4.4%, respectively), but very high prevalence of 21% among goats. Generally, such high rates are observed after a Q fever-related termination of pregnancy as described by Cantas et al. (2011) [30] and Berri et al. (2005) [7]. Indeed, in our study, six small ruminant farms have indicated terminations among pregnant ruminants and our samples were collected within one month after these events. Shedding of *C. burnetii* in vaginal mucus lasts for one to five weeks [31]. In addition, it has been shown that most of the goats that had aborted or delivered normally in naturally infected herds shed the bacteria [32], [33]. Our findings confirmed these previous results because the within-herd bacterial prevalence in the farms that reported pregnancy terminations was estimated to be between 70% and 90%.

This study demonstrates that the risk of Q fever infection of ruminants increased when farms or grazing pastures are in the way of prevailing winds, confirming the airborne route of transmission for *C. burnetii*. In contrast to other studies [34], ticks, which were all detected to be negative for *C. burnetii*, appeared to not be involved in the contamination process. However, the systematic use of deltamethrin may have reduced the tick population and altered their ability to carry *C. burnetii*. Infection of animals or humans and contamination of the environment with *C. burnetii* requires transport through the atmosphere. It is assumed that *C. burnetii* is absorbed or fixed at the aerosol surface and becomes airborne. *C. burnetii* is resistant to heat and dryness and can survive for more than 150 days in the environment. Most ruminants, especially sheep and goats, spend their days grazing outside in the production areas of the highlands or on the eastern coast, where the highest density of ruminants and farms is met and where the winds are blowing most of the year. Additionally, manure is often used as a fertilizer in market gardening in these areas, potentially contributing to the spread of *C. burnetii* [35]. It is notable that contaminated aerosols are a major mechanism whereby *C. burnetii* is transmitted to humans [36]. MLVA genotyping results were in agreement with this risk factor because genotype 5 was observed in five farms located in a 3 km radius of the same area as the eastern windy part of the island [37].

Even if no correlation between pastures exposed to prevailing winds and animals kept at night in their barn was observed, these two variables support the assumption that *C. burnetii* may be transmitted via airborne route. Indeed, our study also showed that the risk of infection for ruminants was lower when the animals were kept in the barn at night. Generally, older cows that stayed in the cow barn for longer periods of time than young animals are more frequently infected. Hence, the probability of being exposed to the bacterium increases with exposure time [38]. However, in Reunion Island, most of the barns, particularly for sheep and goats, are open spaces sheltered from the wind. In these cases, the probability of infection by droplets and aerosols transmitted by wind is lower.

A lack of precautionary measures for visitors (such as washing hands and changing clothes and boots) before entering the farm was also associated with a higher risk for infection of ruminants with *C. burnetii*. The visitors, including veterinarians, food factory staff and professional hoof trimmers, may act as mechanical carriers and transfer the pathogen from infected to non-infected herds. This route of transmission has already been highlighted in previously reported articles [39], [40], suggesting that farm personnel often act as mechanical transmitters of contaminated fomites from an infected herd to uninfected ones.

Conversely, the risk of infection for ruminants was decreased when a proper quarantine was set up before any introduction of new animals to the farm. New ruminants are introduced after purchasing or, in the case of goats, when a male is borrowed from another farm to improve the reproductive performances. We should mention that young goats are often used in religious celebrations on Reunion Island. Again, MLVA genotyping results stressed the risk of infection when no quarantine is set up because, in our study, genotype 7 was detected in only in the two farms that purchased live animals from farm A [40]. A recent study reported that purchase of animals increased the risk of introducing *C. burnetii* infection into cattle herds [23]. This assumption stresses the risk of introduction of the bacteria both biologically and mechanically. Animals that live in close contact can become infected with *C. burnetii* because bacteria are shed from infected animals by vaginal secretions, placenta, urine or feces. A previous study described the occurrence of pregnancy terminations in goat herds that were exposed to three goats from another herd that reportedly kidded prematurely during a fair [41]. Moreover, when cows were imported into an area of endemic infection, 40% of uninfected cows became *C. burnetii*-infected within six months [42]. Viable bacteria have been isolated from sperm of seropositive bulls [43].

Our results demonstrate that even with a relative low seroprevalence in ruminants, *C. burnetii* is circulating consistently in the island. This was particularly evident in goats, where 21% animals were PCR positive. Questions emerged regarding the potential impact of *C. burnetii* on the general population as well as persons at risk, such as pregnant women. Thus, we have begun another study to assess the consequences of this bacterium on human health.
